# Robust imputation-based method for eye, hair, and skin colour prediction from low-coverage ancient DNA

**DOI:** 10.1038/s41598-026-38372-3

**Published:** 2026-02-05

**Authors:** Zoltán Maróti, Emil Nyerki, Tibor Török, Gergely István Varga, Tibor Kalmár

**Affiliations:** 1 Department of Paediatrics, University of Szeged, Szeged, Hungary; 2 Research Centre of Archaeogenetics, Institute of Hungarian Research, Budapest, Hungary; 3 Department of Genetics, University of Szeged, Szeged, Hungary

**Keywords:** Biological techniques, Computational biology and bioinformatics, Genetics

## Abstract

The prediction of externally visible traits (eye, hair, and skin colours) from DNA can provide valuable information for contemporary and ancient human populations. The validated HIrisPlex-S method is the primary tool in forensics for phenotyping modern samples. The HIrisPlex-S multiplex PCR assay can handle trace DNA from modern samples, but the analysis of degraded, low-coverage ancient DNA (aDNA) presents additional challenges. Genotype imputation has recently proven successful in effectively filling in missing information in aDNA sequences. To assess the feasibility of this approach, we evaluated how key factors, such as genome coverage, minor allele frequency, extent of postmortem damage, and the population origin of the test individual, influence the efficiency of imputing HIrisPlex-S markers and predicting phenotypes. We used high-coverage sequence data from modern individuals and ancient remains for the evaluation. Our results demonstrate that even with genome coverages as low as 0.1–0.5×, the proposed workflow can predict phenotypes from degraded ancient (or forensic) whole genome sequence (WGS) data with good accuracy. To aid the archaeogenetics community, we have developed aHISPlex. This user-friendly, easily deployable imputation-based framework includes the new bioinformatics tools and the pre-made reference data sets required for the whole analysis.

**Supplementary Information:**

The online version contains supplementary material available at 10.1038/s41598-026-38372-3.

## Background

The prediction of externally visible human traits from DNA has implications in forensics^1–3^, but it can also provide valuable information for the analysis of archaeological remains. The comparative analysis of some alleles associated with pigmentation led to a deeper understanding of the appearance and spread of some phenotypic traits during the Mesolithic-Neolithic transition in Europe^4–6^ and shed light on the selection affecting eye, skin, and hair colour in the peoples of ancient Eurasia^7^. The most comprehensive and forensically validated HIrisPlex-S system currently uses 41 autosomal markers to predict eye, hair, and skin colour^8–10^. The HIrisPlex-S system was developed and validated using the observed phenotype and genetic data of thousands of contemporary individuals representing a wide range of genetic backgrounds, including European, Asian, and African ancestries. Although the individuals of European ancestry are overrepresented in the HIrisPlex-S validation dataset, the phenotypic variation in light skin tones and blond to brown hair shades among European ancestry is significantly higher compared to darker tones in other ancestries. Consequently, even with this skewed sample distribution, these phenotypes still have the lowest specificity to predict the exact phenotype (0.628–0.767) compared to darker hair or skin tone (typically 0.97–0.98) predictions^11^. While the HIrisPlex-S multiplex PCR assay can cope with trace amounts of DNA in forensic samples, the analysis of ancient DNA (aDNA) presents further challenges. DNA from archaeological remains is prone to postmortem damage (PMD), leading to DNA degradation over time. Typically, aDNA consists of very short DNA fragments, with an average length of 40–60 base pairs, and it undergoes frequent C > T and G > A nucleotide transitions, primarily near the ends of these fragments^12^. These characteristics, including the small average DNA fragment size, potential contamination with high molecular weight modern DNA, and frequent nucleotide changes, render PCR-based methods (including the HIrisPlex-S multiplex PCR assay) unfeasible on ancient samples^13–15^. Consequently, the majority of the population genetic analyses of aDNA are based on shotgun WGS or hybridisation-based targeted methods, as seen in the most comprehensive Allen Ancient DNA Resource (AADR) curated ancient dataset^16,17^.

To date, a substantial number of WGS sequences have been generated from ancient samples to facilitate the genetic characterization of ancient populations. However, due to the persisting challenges in sequencing degraded aDNA, there are currently only a handful of high-coverage WGS (> 10× genome coverage) sequences available from aDNA^18^, while the majority of aDNA samples typically have low genome coverage (0.1×- 2×). Although the current HIrisPlex-S system can handle partial information, its primary reliance on diploid genotype data results in increased uncertainty in phenotype predictions and a higher likelihood of producing invalid results when dealing with missing or low-confidence diploid genotypes. Due to the low genome coverage of aDNA samples, a significant portion of HIrisPlex-S markers typically lacks sequence information. Although sparse pseudo-haploid genotypes from aDNA can be used in several population genetic approaches like PCA, admixture, and qpAdm^19,20^, this data is not suitable for the HIrisPlex-S system, as it relies on the diploid genotypes to predict the most probable phenotypes.

With the increasing number of low-coverage WGS sequences, new tools and approaches are emerging to address the challenges of analysing incomplete data. One of the latest approaches is to impute the missing diploid genotypes/haplotypes from partially genotyped data. This approach relies on the observation that closely linked markers tend to be inherited together as haplotypes from parents to offspring. Hence, a catalogue of such common haplotypes and the partial information on sequential stretches of markers can be used to predict the most likely diploid state of markers in the low-coverage sample. Several algorithms and tools have been developed to balance speed and precision in addressing the large computational requirements of the imputation^21–23^. Available literature indicates that imputation precision depends on multiple factors, including the fraction of missing and genotyped markers, the minor allele frequency of the imputed marker, the density of genotyped markers, the reference data set, the genome structure/population origin of the imputed sample, and the choice of algorithm/tool^8–10^.

State-of-the-art imputation tools can achieve high genotyping precision for common variants with high minor allele frequency (MAF), even when dealing with partial information corresponding to 0.5× genome coverage data^24^. This suggests that genotype imputation could be applied to allow phenotyping ancient individuals from aDNA. While a recent manuscript has shown that imputed diploid genotypes have high overall concordance even from noisy, low-quality, and low genome coverage ancient samples^18^, there is currently no data available to assess whether this approach can reliably be used for evaluating complex phenotypic traits in ancient samples. Since most genetic markers of eye, hair, and skin colours consist of common alleles with high MAF, imputation has the potential to predict the diploid genotype state of these markers even from low-coverage aDNA. Given that the three predicted phenotypic traits result from the combination of several trait-defining markers, we hypothesised that a few imputation errors would not significantly impact the prediction of the most likely phenotype.

Another challenge is that the entire workflow, from aligned NGS data to phenotypic classification, involves numerous tools, data preparation, and data shaping, with no single straightforward tool available to perform the entire analysis. Consequently, our goal was to create a user-friendly tool to facilitate the imputation-based workflow and to evaluate the effect of the major influencing factors on the proposed workflow. The proposed framework contains the new bioinformatics tools to translate the reference-based genotypes to the probe-based allele counts, evaluate the p-values of the HIrisPlex-S system, and predict the phenotypes based on the rules described in the HIrisPlex-S user manual, including the pre-made reference data sets required for the analysis. In the workflow, we selected the last version of the GLIMPSE tool (GLIMPSE2, version 2.0.0) for imputation with the gold standard phased 1 KG reference data, as it promises very fast and accurate imputation compared to other tools^25,26^. Using our framework, we assessed the effect of major influencing factors (the genome coverage, minor allele frequency, extent of PMD, and the population origin of the test individual) on the imputation efficiency and phenotype prediction using high-coverage experimental data from modern individuals and ancient remains.

## Results

Imputation was performed on the 1.7 million common variants within the 11 genome segments encompassing the 41 HIrisPlex-S markers, covering a total of around 70 megabases, as described in the methods section. With the provided tools, we filtered and translated the genotypes of the 41 markers from the reference-based data to probe/allele count data, conforming to the requirements of the HIrisPlex-S web tool to evaluate the predicted phenotype probabilities.

### Validation of imputation accuracy on simulated modern data

We included 93 unrelated modern samples from the 1 KG Phase 3 data with diverse ancestry and phenotypes representative of all observed phenotype variations in each 1 KG population (see the Method section for the selection criteria). For the validation of the imputation accuracy of the HIrisPlex-S markers, the original high-coverage data were downsampled to 10×, 2×, 1×, 0.5×, and 0.1× genome coverages in 10 parallel replicates, each with different random seeds. In this analysis, we used a custom reference data set where all selected individuals and their relatives were excluded from the GLIMPSE2 binary reference data to avoid imputation bias. We filtered the genotypes from the gold standard high coverage 1 KG Phase 3 VCF data set for these individuals and also the imputed genotype data from the down-scaled data set. Since the HIrisPlex-S system uses probe-based allele counts that are not directly compatible with the reference-based genotypes (depending on whether the PCR probes were designed on the forward or reverse strand), we implemented a tool and created the translation tables for each human reference (hg19, GRCh37, GRCh38, hg38). With this tool, the reference-based genotype (GT) data were translated to the HIrisPlex-S marker counts, and the HIrisPlex-S web service was used to calculate the 14 p-values for the predicted phenotype classification. For easy usage, we implemented a tool to turn the p-values to the most probable phenotype classifications based on the rules described in the HIrisPlex-S system user manual (Supplementary Table S1).

### Imputation accuracy of the HIrisPlex-S markers in simulated modern data

We used the curated 1 KG Phase 3 VCF data as ground truth to evaluate the genotyping accuracy of imputation at different genome coverages. We distinguished between two types of genotyping errors based on their potential impact on phenotype prediction. The ‘total error’ encompassed all cases where the imputed diploid genotype differed from the ground truth. The ‘opposite error’ refers to instances where homozygous opposite alleles appeared in the imputed sequence compared to the truth (HOM REF instead of HOM ALT or vice versa), as the second category would more severely affect the phenotype prediction probabilities, considering either dominant-recessive or quantitative inheritance. We included the result of all imputations with population data and phenotype classification in Supplementary Table S1. We summarized the mean imputation error rate for the different super populations and the whole validation data set at each different genome coverage (Table 1).

**Table 1:** Mean imputation error rates observed in the different super populations of simulated modern data based on 5× 10 parallel imputations of the 93 selected individuals at different genome coverages; ALL, all super populations; AFR, African; AMR, Ad Mixed American; EAS, East Asian, EUR, European; SAS, South Asian origin.

Super population	N	Mean imputation genotyping error fraction (%)									
		Total error					Opposite error				
		0.1× (%)	0.5× (%)	1× (%)	2× (%)	10× (%)	0.1× (%)	0.5× (%)	1× (%)	2× (%)	10× (%)
ALL	93	4.96	1.17	0.68	0.35	0.02	0.09	0.01	0.00	0.00	0.00
AFR	9	2.20	0.41	0.14	0.05	0.00	0.00	0.00	0.00	0.00	0.00
AMR	19	6.24	1.41	0.80	0.47	0.03	0.10	0.00	0.00	0.00	0.00
EAS	20	3.27	0.74	0.43	0.20	0.01	0.04	0.01	0.00	0.00	0.00
EUR	25	5.59	1.63	1.00	0.50	0.02	0.10	0.00	0.00	0.00	0.00
SAS	20	5.88	1.15	0.67	0.33	0.01	0.15	0.01	0.00	0.00	0.00

Although any imputation error (indicating a false HET instead of HOM REF or HOM ALT) may lead to invalid phenotype classification in theory, the largest effect would be due to falsely suggesting the opposite genotype of the HOMALT or HOMREF case, as in any inheritance model, this would cause the maximum effect on the predicted phenotype. As shown, the opposite error fraction was very low (less than 0.15 percent), even at 0.1× genome coverage. The total genotyping error rate was below 2%, even at 0.5× genome coverage. Even at 0.1× genome coverage, it was not higher than 5–6% in the case of Ad Mixed Americans, Europeans, and South Asian origin people.

**Fig. 1:**
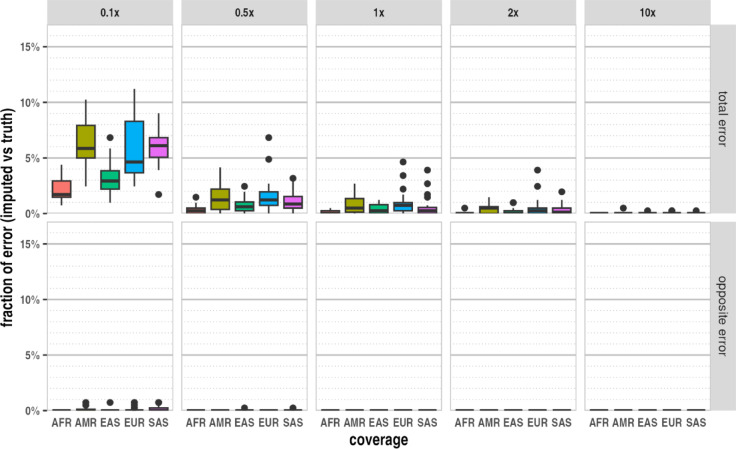
The distribution of imputation error of the 93 selected representative 1 KG individuals in the different super populations at different genome coverages: ALL—all super populations, AFR—African, AMR—Ad Mixed American, EAS—East Asian, EUR—European, SAS South Asian origin.

The distribution of the imputation error (Fig. 1) within the individuals revealed that at 0.5× or higher genome coverage, the majority of individuals had consistently low imputation error rates. However, in the case of EUR and SAS super populations, a few individuals had higher (1–7%) imputation error rates even at high genome coverages, suggesting reference error.

The error rates show that even as low as 0.1× genome coverage, where we have haploid marker information at about 10% of our genotyped markers, imputation still recovers the diploid state of the 41 markers, and statistically only 2–3 markers are predicted wrong (and even in the case of imputation errors, the opposite error is almost non-existent). We also extracted the imputation accuracy metric from the GLIMPSE2 *bcf* files (Supplementary Table S2) to test whether any marker has very low values. However, our data suggests that even at 0.5× genome coverage, the imputation quality of the HIrisPlex-S markers is very high (> 0.99), and only a few rare MC1R markers had lower values.

### Imputation accuracy of the MC1R markers

While most HIrisPlex-S markers have high minor allele frequencies, the less common red hair trait is associated with low minor allele frequency genetic variations^27^. The HIrisPlex-S system contains 11 MC1R markers that majorly influence the prediction accuracy of red hair traits (the 0.92 AUC of red hair is dropped to 0.65 AUC when the genotypes of all 11 MC1R markers are excluded, and in this case, the HIrisPlex-S system does not predict hair phenotype at all). Seven of the MC1R markers (rs312262906, rs11547464, rs1805008, rs1805006, rs1805009, rs201326893, and rs1110400) have low (< 1.5%) global MAF. Furthermore, unlike all other HIrisPlex-S markers, the lowest global MAF (0.00078) red hair-associated rs312262906 MC1R marker is a single base pair duplication (NC_000016.10:g.89919344dup) that is technically very hard to predict by the multiplex PCR assay^10^. It is also known that low MAF rare variants have worse imputation accuracy^18,26^. The typically lower genotyping accuracy of small insertions or deletions compared to unique SNPs, coupled with the low MAF, could potentially have a significantly adverse impact on the genotyping and imputation accuracy of these markers. Accordingly, we included 8 red-haired individuals (each with a different MC1R variant) to test whether these particular phenotypes influencing rare variants have lower imputation accuracy compared to the high MAF HIrisPlex-S variants.

We collected all genotyping errors in the 11 MC1R markers for the 8 red-haired individuals and also for the non-red-haired individuals to assess the false negative and false positive imputation error rate of these variants in our validation data set.

According to our data, the false negative rate of mis-genotyping an MC1R mutant to a homozygous REF allele is comparable with the imputation accuracy of higher MAF HIrisPlex-S markers (Tables 1, 2). Furthermore, the false positive rate of non-red-haired individuals with invalid imputation in the 11 MC1R variants was also low. We have to note that some of the included MC1R variants have very high global MAF (like rs885479 with MAF 0.183; rs2228479 with MAF 0.0766) that is incompatible with the global red hair phenotype frequency, thus they are likely only in linkage with low MAF true red hair variants in a subset of red hair individuals. Consequently, a genotyping error in such MC1R markers does not always lead to a falsely indicated red hair phenotype.

**Table 2:** The mean genotyping error rates of the 11 MC1R markers in the red hair and non-red hair indicated 1 KG individuals based on 5× 10 parallel imputations of the 93 selected individuals at different genome coverages.

Individuals	N	Mean imputation genotyping error fraction (%) of 11 MC1R markers									
		Total error					Opposite error				
		0.1× (%)	0.5× (%)	1× (%)	2× (%)	10× (%)	0.1× (%)	0.5× (%)	1× (%)	2× (%)	10× (%)
Red haired	8	2.95	1.48	0.68	0.23	0.00	0.00	0.00	0.00	0.00	0.00
Non-red-haired	85	1.84	0.62	0.49	0.33	0.01	0.00	0.00	0.00	0.00	0.00

To investigate the effect of genotyping errors in the MC1R markers, we calculated the ratio of red hair individuals classified as non-red hair individuals (false negative red hair detection rate) and the ratio of non-red hair individuals classified as red-haired individuals, comparing the classification based on the truth and the imputed genotypes at various coverages (Table 3).

**Table 3:** The misclassification rate of non-red-haired and red-haired individuals in the modern data set is based on the comparison of truth with the imputed data of 85 non-red-haired and 8 red-haired individuals.

Coverage	FP red hair (N = 850) (%)	FN red hair (N = 80) (%)
10×	0.0	0.0
2×	0.5	6.25
1×	0.8	15.0
0.5×	0.8	22.5
0.1×	0.2	38.7

Our results showed that the misclassification of red-haired individuals as mainly blond or dark blond individuals is elevated (~ 6% false negative rate) even at 2× genome coverages (Table 3); hence, this hair trait is less reliably identified. However, the majority of cases are still properly classified as red, even at 0.1× coverage. The false positive rate of indicating a non-red-haired individual as red-haired was very low. Our data revealed that only one blue-eyed, blond/dark blond-haired and intermediate (lighter) skinned individual (NA20792, TSI, EUR) was classified invalidly as red-haired in our whole data set. The genotype imputation error always occurred at the same two MC1R markers (rs1805008 and rs1805006), where instead of the HOM REF allele, a heterozygote ALT allele was imputed. This imputation genotype error leads to falsely indicating blue eyes, red hair, and pale (lighter) skin phenotype in these cases. Since this genotyping error was occurring only in one individual but was present also in 2×, 1×, 0.5×, and 0.1× genome coverages as well, it is likely that the imputation error is due to insufficient reference data available for individuals with similar genome structure at the MC1R locus. We have to note also that, to our surprise, the GRCh38 gold standard common allele joint VCF data did not include a very rare MC1R marker compared to the GRCh37 joint VCF. In particular, the GRCh38 reference data lacks the rs201326893 STOP mutation for the HG01204 sample that exists in the GRCh37 JOINT VCF. We tested, that the GRCh38 alignment file (HG01204.final.cram) at this position do have this variant in* “samtools mpileup”* in the proper heterozygous state (C/A genotype with 20/15 allele counts respectively), hence it is likely that the common allele criterion between the two reference joint VCF file likely changed and this very rare allele was not included in the GRCh38 data set. We also have to note that out of the 24 red-haired individuals, we had to exclude the 8 red-haired test individuals from the reference data used for the imputation. Consequently, in our validation experiments, we significantly worsen the imputation accuracy of red hair-associated markers (especially for the rare variants existing in only very few individuals). We suspect that the significant thinning of the reference data for the MC1R variants is the cause of generally lower MC1R marker qualities reported by GLIMPS2 for modern individuals (Supplementary Table S2) as compared to other markers. Consequently, the analysis with the full reference set should perform better in real-world usage with the complete reference data, and our analysis leads to improved FP/TP figures for red hair individuals compared to our validation data set. The absence of rs201326893 from the GRCh38 reference data poses a limitation on analysing modern data based on this reference, as this marker is not imputed.

### Evaluation of the aHISplex method on experimental high-coverage ancient samples

We selected all of the 31 available high coverage (> 10× genome coverage) ancient WGS shotgun sequences from the Allen Ancient Data resource (AADR, version 54.1)^17,28^. The sample IDs, the associated annotations from the AADR database, and the global population structure based on the fastNGSadmix tool^29^ of the selected samples are found in Supplementary Table S3. From the high-coverage WGS data, we obtained the most likely genotypes of the 31 samples for all 41 HIrisPlex-S markers, except for a few positions where they had 5× or lower genome coverages (Supplementary Table S4). We utilized the diploid genotypes from the high-coverage data as the ground truth for assessing concordance and genotyping errors in imputation.

In the case of ancient data, we conducted 10 parallel imputations for each downsampled dataset, resulting in a total of 100 parallel imputed sets of genotypes for each sample at each simulated genome coverage (10×, 2×, 1×, 0,5× and 0.1×). We filtered the imputed genotypes of each 41 HIrisPlex-S marker from all imputed variants and compared them with the ground truth to calculate the fraction of imputation errors. As imputation is a probabilistic method, and genotyping errors due to postmortem damage in ancient DNA could influence the posterior probabilities of the most likely haplotypes, we also tested the concordance of the imputed genotypes from the original high-coverage data and the ground truth. Accordingly, we also imputed the diploid genotypes from the original high-coverage samples in 100 parallel runs (Supplementary Table S5).

### Effect of genome coverage on imputation accuracy in ancient samples

**Fig. 2:**
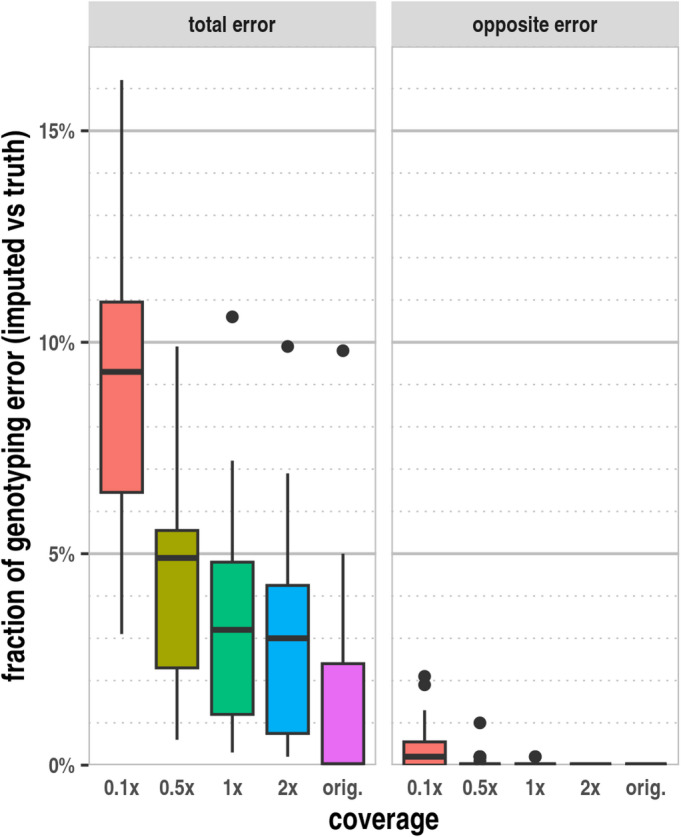
The effect of genome coverage on the mean imputation error based on the analysis of 100 parallel imputations of 31 ancient samples. In the “total error” category, any imputed genotype that did not match the expected ground truth was considered. In the “opposite error” category, we only considered cases where homozygous opposite alleles appeared in the imputed sequence compared to the truth.

According to our results, the mean imputation error rate was most severely affected by the genome coverage (Fig. 2), as this factor influences the number/density of genotyped markers used to predict the most likely haplotype configurations and diploid genotypes at all loci.

The accuracy of the imputed diploid genotypes is noteworthy. The median concordance of imputed diploid genotypes with the truth is approximately 95% at 0.5× and approximately 91% at 0.1× genome coverage for the analysed samples. At higher genome coverages, the accuracy increases up to 97%. Nevertheless, even in the original high coverage data, a very low frequency of imputation errors can be observed, likely at variants with skewed allelic balance. The rate of opposite genotype errors is negligible at or above 0.5× genome coverages, and even at 0.1× genome coverage, only a few samples had more than 1% of this type of error. At 1× genome coverage and below, most markers are usually represented by a single read, with only one allele randomly sampled. At 0.5× genome coverage, statistically half of the markers are covered, nevertheless, the overall diploid genotyping accuracy remains 95% or higher. This high accuracy demonstrates the feasibility of imputing diploid genotypes for the HIrisPlex-S markers from ancient, low-coverage shotgun WGS data.

### The effect of sample age, population origin and genotyping errors on imputation accuracy

We also calculated the error rates in different sample subgroups to assess other factors that can impact imputation accuracy. These factors included sample age (which affects postmortem aDNA damage), the use of Uracil*-*DNA glycosylase (UDG) treatment (as it drastically reduces the amount of transition errors in the sequences), and the sample’s source population(s) (as the reference data and the genome structure of the test population could be largely different). Unfortunately, the number of available high-coverage ancient samples does not allow us to create homogenous groups based on all potentially influencing factors. Therefore, we created contrasting subsets of samples (containing at least three samples) that differed in one or more influencing factors. We then calculated the mean genotyping error rates for these subsets (Table 4).

**Table 4:** Mean imputation error rates observed in the different subsets of samples at different genome coverages based on 100 parallel imputations.

	N	Mean imputation genotyping error fraction (%)									
		Total error					Opposite error				
		0.1× (%)	0.5× (%)	1× (%)	2× (%)	orig. (%)	0.1× (%)	0.5× (%)	1× (%)	2× (%)	orig. (%)
ALL	31	9.0	4.4	3.4	2.9	1.4	0.4	0.1	0.0	0.0	0.0
no UDG (ALL)	15	9.2	4.4	3.6	3.1	1.9	0.4	0.1	0.0	0.0	0.0
UDG (ALL)	16	8.7	4.4	3.3	2.8	0.9	0.5	0.0	0.0	0.0	0.0
EUR (noUDG)	3	11.4	6.6	5.4	3.8	1.6	0.4	0.0	0.0	0.0	0.0
EUR (UDG)	9	9.5	4.1	2.5	2.1	0.5	0.6	0.0	0.0	0.0	0.0
AFR (UDG)	3	7.6	6.0	6.0	5.4	3.3	0.3	0.1	0.1	0.0	0.0
30,000–41,400 BP	3	10.4	4.9	4.0	3.3	0.8	0.8	0.2	0.1	0.0	0.0
8240–9615 BP	4	8.6	2.8	2.4	1.4	0.9	0.1	0.0	0.0	0.0	0.0
163–1170 BP	5	6.5	4.7	4.3	3.6	2.0	0.3	0.1	0.0	0.0	0.0
EUR (ALL)	12	9.9	4.7	3.2	2.6	0.8	0.5	0.0	0.0	0.0	0.0
AFR (ALL)	4	8.0	5.6	5.5	5.0	4.9	0.3	0.1	0.1	0.0	0.0
OTHER (ALL)	15	8.5	3.9	3.0	2.6	0.9	0.3	0.1	0.0	0.0	0.0

UDG, Uracil*-*DNA glycosylase; EUR, European origin; AFR, African origin; BP, before present.

The detailed data of the imputation genotype error rates for each sample at the different genome coverages are shown in Supplementary Table S6. Overall, the mean error rates in the selected subgroups exhibit only minor differences, underscoring that the primary factor influencing imputation errors is the genome coverage. It is noteworthy that samples of African origin (AFR) exhibit higher imputation error at higher genome coverages, even when using the original high-coverage data. This observation strongly suggests the presence of reference errors, stemming from the fact that the greater haplotype diversity among AFR populations is not adequately represented in the 1 KG reference panel. It appears that UDG treatment slightly decreases the imputation error rate, as evidenced by the difference between UDG-treated and non-UDG-treated individuals of European origin (EUR). This effect is likely associated with the higher level of PMD in samples without UDG treatment, which leads to an increased rate of random C > T and A > G transitions in ancient DNA sequences. We also observe a slight increase in the mean imputation error among older dated samples. As sample age in general correlates with the PMD level in aDNA, this increase in imputation error rates is likely attributable to the age factor. Importantly, the opposite genotype error rate remains negligible at 0.5× genome coverage levels or higher; even in the oldest samples at 0.1× genome coverage, the opposite error rate did not exceed 0.8%.

### The imputation accuracy of the individual HIrisPlex-S markers in ancient samples

To evaluate whether certain markers exhibit higher imputation error rates due to low MAF, insufficient linked marker context, or poorly represented haplotypes in the reference data, we also calculated the mean genotyping error rate for each marker (Supplementary Table S7). Overall, most of the 41 HIrisPlex-S markers, including those with lower MAF, had good imputation accuracy. Nevertheless, we noticed that certain markers had markedly worse imputation accuracy in specific samples, even at high genome coverages.

To distinguish between high and low imputation error rates, a threshold value needs to be defined. We established this threshold based on the currently available imputation accuracy for African genomes. In the literature, the highest imputation error rates (> = 15%) are observed in African samples, even at higher genome coverages. Such systemic imputation errors by reference panel-based imputation tools could occur because the haplotype dictionary created from the high-quality reference data may not represent all existing haplotypes. Furthermore, since the reference panel is based on sequences of modern individuals, a subset of markers (especially rare alleles) did not yet exist in ancient individuals because the founder mutation occurred at a later time. Imputation will still yield the overall best-fitting haplotype even in these cases; however, specific SNPs missing from the reference panel or from the ancient individuals may be systematically biased. This type of imputation error we refer to as reference panel bias^30,31^, to distinguish from random imputation errors caused by low genome coverage (missing genotype data), which could also lead to improper ascertainment of the true diploid genotypes.

In our analysis, we considered the mean imputation error at high genome coverages (original, 2×, and 1×) exceeding 15% as “high” error rates and those below this threshold as “low” error rates. Applying this criterion, we identified 55 sample/marker combinations that had significantly elevated imputation error rates across the original and all lower coverage imputations (Supplementary Table S8). Out of the total of 1271 sample/marker combinations (31 genomes with 41 markers), this small subset of combinations accounted for ~ 47% of all imputation errors, as indicated by the number of imputation errors in the second row of the high error rate category in Table 5.

**Table 5:** Summary of high and low error rate sample/marker combinations. The criterion of >  = 15% mean imputation error frequency observed in the high genome coverage (original, 2×, 1×) imputations was used to distinguish between high/low imputation error rate combinations.

Genome coverage	High error rate sample/rsID combinations (N = 55)*					Low error rate sample/rsID combinations (N = 1217)				
	Original	2×	1×	0.5×	0.1×	Original	2×	1×	0.5×	0.1×
Number of imputed genotypes	5500	5500	5500	5500	5500	121,600	121,600	121,600	121,600	121,600
Number of imputation errors	1748	2470	2735	2818	2793	0	1219	1618	2814	8610
% of imputation errors (error/no. of imputations*100)	31.78%	44.91%	49.73%	51.24%	50.78%	0.00%	1.00%	1.33%	2.31%	7.08%
Fraction of total error in the given category (high/low error rate vs total error)	100.00%	66.96%	62.83%	50.04%	24.49%	0.00%	33.04%	37.17%	49.96%	75.51%

Imputation determines the best likelihood of all genotype data within a large chunk of the analysed genome region of the test individual that fits the best-matching diploid allele combination inferred from the reference data. Discrepancies between linked markers in the sample and linkage information in the reference data can lead to inevitable ambiguities or incorrect predictions of the diploid genotype. We extracted the imputation quality values from GLIMPSE2 phased/imputed *bcf* output for the HIrisPlex-S markers (Supplementary Table S2) to test for potentially weak regions/markers (with less amount informative, linked markers or contradicting genotypes in the haplotype due to contamination or PMD). Although the imputation qualities were slightly lower than observed in modern data, most of the markers still had very high (> 0.98) values even at 0.1× genome coverage and in the case of the very low MAF MC1R markers. Our data suggests that within this smaller subset of sample/marker combinations, the primary cause of the elevated error rate is likely due to reference panel bias. Most prominently, imputation error occurs even in the original high coverage data where the imputation quality metrics were close to 1. Furthermore, within the high-error category, the overall frequency of imputation errors remains significantly elevated at every level of genome coverage. This is in contrast to the low-error category of sample/marker combinations, where errors tend to increase as genome coverage decreases (3rd row in Table 5).

### Phenotype classification accuracy of the ancient samples

The HIrisPlex-S system calculates the probabilities of each phenotypic trait using a model that was cross-validated on a large contemporary dataset comprising the genotype information of the 41 markers and the known phenotypic information. The web tool translates the genotype information of markers into 14 p-values^32^, which can subsequently be used to classify each phenotypic trait using a complex decision tree described in the HIrisPlex-S user manual. Due to the varying number of phenotype-associated markers and the HIrisPlex-S classification scheme, the number of phenotype categories differs for the three phenotypic traits. The classification of the eye phenotype (blue, brown, black) is straightforward, as the highest phenotype probability (p blue eye, p brown eye, p black eye) indicates the most likely eye colour. In contrast, the classification of hair colour (red, blonde, dark-blonde/blonde, dark-blonde/brown, brown, dark-brown/brown, dark-brown/black, black) and especially the skin phenotype (very pale, very pale/darker, pale/lighter, pale, pale/darker, intermediate/lighter, intermediate, intermediate/darker, dark/lighter, dark, dark/dark-black, dark-black) is more complex. It relies on a greater number of phenotype-associated markers and involves complex heuristic rules based on the calculated p-values, as described in the HIrisPlex-S web tool manual^11^. To simplify and streamline phenotyping, we have incorporated a tool in our software package (*classifHISplex*), which classifies the three phenotypic traits based on these rules and the p-value output file from the HIrisPlex-S web tool.

**Fig. 3:**
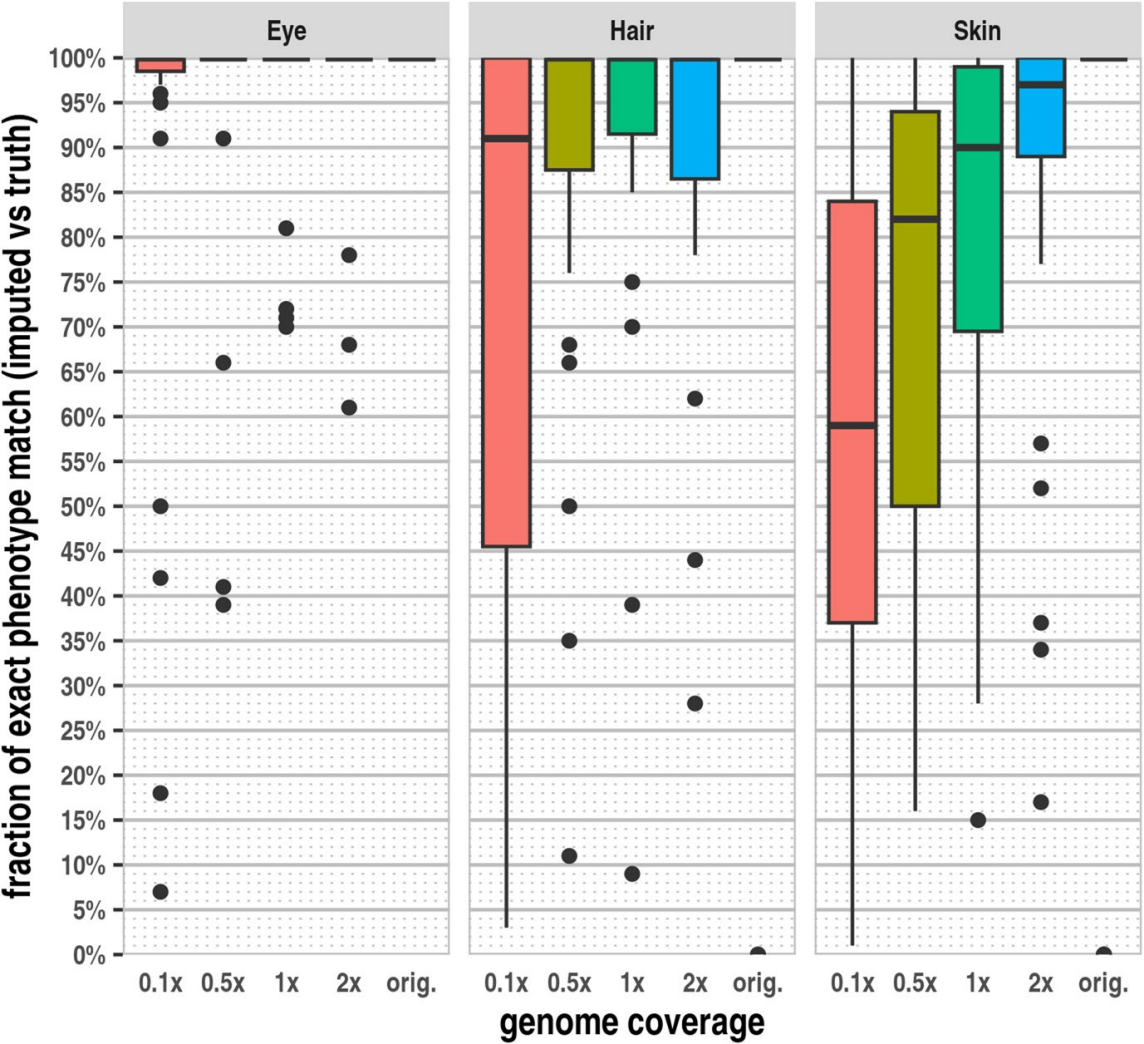
The fraction of exact matching predicted phenotypes in the ancient dataset for the three phenotypic traits. The phenotypes based on the genotypes of 41 HIrisPlex-S markers from the original high-coverage data were used as the ground truth for each sample. We compared the phenotypes predicted from the genotypes of the high-coverage data with those based on the 100 parallel imputated genotypes and calculated the ratio of exact matching phenotypes for each sample at each genome coverage.

To evaluate the impact of imputation errors on phenotype classification, we conducted a comparison between classifications based on the genotypes from high-coverage data (considered as ground truth) and those based on the imputed genotypes at various genome coverages for each modern and ancient sample (Supplementary Tables S1, S5). In general, phenotypic traits with a smaller number of phenotype-associated markers tend to result in statistically fewer imputation errors but potentially have a greater effect on the predicted phenotype, whereas a larger number of phenotype-associated markers tend to lead to statistically more imputation errors but with less drastic changes in the phenotype classification. For both eye and hair phenotypes, the majority of parallel imputations yielded an exact matching phenotype classification with the ground truth, even at 0.1× genome coverages (Fig. 3).

The fraction of exact matching skin phenotypes was lower, particularly at lower genome coverages and higher imputation error rates. Although the fraction of exact phenotype classifications is lower in the case of the skin phenotype, even at higher imputation error rates, a spectrum of very similar predicted phenotypes, compared to the ground truth, is indicated. Meanwhile, the number of significantly different phenotypic classifications remains relatively low. Our data suggests that this is primarily due to the finer granularity of classification in the case of the skin phenotype. Our results demonstrate that the observed rate of imputation errors, even at very low genome coverages, has a relatively minor impact on the phenotype probability values and the classification of intermediate phenotypes. However, for the extreme light complexity of the phenotypic traits where multiple genetic variations are in the homozygous “no pigmentation” state, such as blue eye, blond hair or very pale skin, an imputation error, even in one of the underlying homozygous markers, leads to darker phenotype and a more severe misclassification. While we had only one individual with dark blonde hair and no individuals with very pale skin among the high genome coverage ancient samples, five out of the 31 samples were classified as having blue eyes. In these samples, the concordant blue eye phenotype was predominantly indicated in the majority of parallel imputations at genome coverage 1× or higher. For two samples (Loschbour.DG and VK1_noUDG.SG), the majority of imputations yielded the expected blue eye phenotype (91% and 100%, respectively) even at 0.5× genome coverage. In contrast, for the other three samples (vbj004_noUDG.SG, SF12.SG and SZ45.SG) at 0.5× genome coverage, the blue eye phenotype was misclassified as brown with a high probability (61% and 59%, 34% respectively, Supplementary Table S5). As expected, the misclassification rate of the blue eye phenotype was even higher at 0.1× genome coverage. When investigating the eye phenotype-associated markers, our data revealed that the higher-than-expected rate of imputation error in latter samples was attributed to sample (population) specific reference panel bias of the rs12913832 marker (Supplementary Table S8). The three particular samples (vbj004_noUDG.SG /Sweden Gotland Vasterbjers Pitted WareBattleAxe/, SF12.SG /Sweden Mesolithic/, SZ45.SG /Hungary Langobard outlier/) likely share similar marker combinations in this locus, which are poorly represented in the reference dataset. In these particular samples, the homozygous A/A minor allele is imputed as heterozygous A/C in approximately 30% of the cases, even at 1–2× genome coverages, signifying reference panel bias.

## Discussion

Genome-wide association studies (GWAS) not only identified genotypic variations that influence disease susceptibility, but also identified several genetic loci responsible for defining visible phenotypic traits^33–35^. Accordingly, the genotype information could be used to predict these traits when only the DNA is available from the subjects. One such case is forensic analysis, where the HIrisPlex-S system was developed and validated using a large cohort of contemporary individuals of diverse origins (European, Asian, and African) with known phenotype and genotype information^8–10^. While the method is suitable to work even from trace amounts of biological material, its usage is restricted to modern samples with high molecular weight DNA due to the limitation of the PCR-based approach. Another field where DNA-based phenotyping could lead to new insight is archeogenetics, where a large number of ancient DNA sequences are being generated to study the population genetics and evolutionary processes of our ancestors. Scientific facial reconstruction could also benefit from objective DNA-based approaches. For example, the phenotype data could lead to better visualisation of historical figures when historical records are scarce or ambiguous.

The available pseudo-haploid genotype data from ancient individuals are suitable for tracking the evolution of phenotype-related loci to test for the existence of specific variants in ancient populations. Analysis of 170 skin pigmentation-related markers ascertained in the UK Biobank showed that, in a large cohort of ancient samples (including very old samples), selection of light skin pigmentation-related markers was driven by a relatively small proportion of large-effect variants that are associated with present-day phenotypic variation^36^. These results suggest that analysis of the major phenotype-associated loci (as the HIrisPlex-S markers) could be theoretically used to predict the visible traits of ancient individuals. However, the analysis of low coverage, degraded ancient DNA poses challenges for proper diploid genotype assessment, a requirement for accurate geno-phenotype predictions. Consequently, despite the abundance of available aDNA sequences, we have phenotypic information only for a handful of ancient samples.

Imputation of the most likely haplotypes from sparse pseudo-haploid data promises to uncover the diploid genotypes with high confidence. While a very recent manuscript has shown that imputed diploid genotypes have high overall concordance even from noisy, low-quality, and low genome coverage ancient samples^18^, there is currently no data available to assess whether this approach can reliably be used for the evaluation of complex phenotypic traits in ancient samples. Another challenge is that the entire workflow, from aligned NGS data to phenotypic classification, involves numerous tools, data preparation, and data shaping, with no single straightforward tool available to perform the entire analysis. Consequently, our goal was to create a user-friendly tool to facilitate the imputation-based workflow and to evaluate the effect of the major influencing factors on the proposed workflow. Accordingly, we investigated how the genome coverage, genotyping errors, sample age and the overall genome structure (population origin) of the sample influence imputation errors and the accuracy of phenotype prediction.

There are different approaches used for imputation^21–23^; however, the majority of imputation tools infer the common haplotypes from phased, fully typed diploid references. While some approaches can assess common haplotypes 'on the fly’ using a vast number of jointly analysed samples, these tools typically require very large sample sets and higher CPU resources. Unfortunately, such large, high-quality sample sets are not yet available for ancient samples. Therefore, despite their limitations, reference-based imputation methods appear to be the only feasible option for analysing ancient samples. The imputation accuracy of reference panel-based methods inevitably depends on the reference used, as the representation of common haplotype configurations (series of linked markers) serves as a model to impute the most likely haplotypes of the test sample. The assumption that our gold standard reference data set represents most of the likely haplotypes in our test data imposes limitations on the approach. Consequently, imputation can potentially yield false haplotypes/genotypes that exist in our reference but not in the test data. This can occur, for example, in the case of young markers that did not exist yet at the given date in the ancient sample or when the genome structure of the test individual is not (properly) represented in the reference dataset.

To assess the applicability and robustness of the whole workflow, we classified all 1 KG individuals by the HIrisPlex-S system using the gold standard 1 KG genotypes for the 41 markers. Next, for each population, we selected a subset of individuals to represent all of the different phenotypic variations of each of the three phenotypic traits existing in the particular population. Furthermore, we also added 8 red-haired individuals carrying different genetic variants of the MC1R markers to our validation data set. To test the accuracy of imputation, the high coverage sequence alignments of these 93 representative 1 KG individuals were downsampled to 10×, 2×, 1×, 0.5×, 0.1× genome coverages in parallel, and the downsample data was imputed. The original high-quality 1 KG genotypes and predicted phenotypes were compared with the imputed genotypes and the phenotype classification based on the imputed genotypes (Supplementary Table S1). Our results show that the mean of imputation errors were very low (0.41–1.63%) even at 0.5× genome coverage and only significantly increased (2.2–5.8%) at 0.1× genome coverage (Table 1). Our analysis revealed minor differences in the mean imputation error rates among different populations. Previous literature has shown that the 1 KG phase 3 reference data set is less representative for populations of African origin, leading to a higher imputation error rate^18,23,25^. The mean imputation error rate at 0.5× genome coverage, considering all variants in whole genome sequences, is expected to be 15% or higher for genomes of African origin^18^. Surprisingly, individuals with African origin exhibited decreased imputation error rate (0.41%) at 0.5× and even at 0.1× (2.2%) genome coverages. It is known that many markers associated with pigmentation are under selection pressure in Africa^37,38^, leading to less genetic diversity. Consequently, the fewer common haplotypes of AFR origin people around these loci are likely better represented in the 1 KG reference. Our results also showed that at 0.5× genome coverage, the very low MAF red hair-associated MCR1 gene markers have a comparable genotype error rate (~ 0.62–1.48%) as the overall genotype (GT) error rate of the 41 markers (Table 2). Accordingly, the false positive read hair identification is extremely low (0.2–0.8%). On the other hand, the false negative rate of red hair identification is higher, as even a single wrongly imputed marker would suggest mainly blond or dark blond hair instead of the expected red hair (6.25–38.75%; Table 3). In summary, our results suggest that the proposed method is robust as the representative 93 1 KG individuals, reflecting the comprehensive range of phenotypic variations present in each 1 KG population, had very low imputation error rates.

Since postmortem damage and potential exogenous contamination and reference panel bias due to the date of samples and the SNP fixation in time may also interfere with the accuracy of imputation, we also evaluated our method with 31 high-coverage ancient data, where the sequencing depth allowed for diploid calling and phenotyping. As with modern data, we also downsampled the high-coverage data and applied imputation to predict the diploid genotypes from low-coverage experimental data. Our results revealed that, as in the case of modern data, the imputation error rate is mainly the factor of genome coverage. Our analysis revealed that, unsurprisingly, ancient data had a higher imputation error rate compared to modern data (Table 4, Supplementary Table S7). Our analysis also suggests that a portion of the imputation error is not random. Specifically, we identified 55 high error rate ancient sample/marker combinations that collectively accounted for approximately ~ 47% of all imputation errors. Our results indicate that only specific ancient samples had very high imputation errors at specific markers, even at the original, 2×, and 1× genome coverages, while the rest of the samples had very low error rates for these markers (Supplementary Table S8, Table 5), comparable with the figures seen in modern data. In very old ancient samples, a smaller portion of markers (markers that were fixated or widespread at a later date) may be absent compared to 1 KG modern reference genotypes. Since imputation of the already fixated, high MAF markers is also based on the observed pattern of the spatial genetic context around each marker, such differences between the reference haplotype dictionary haplotypes and the observed subset of genotypes/haplotypes of the ancient data may lead to increased uncertainty and imputation errors. When the reference is not representative of the haplotype combination of specific samples, we can expect a high deterministic imputation error. Our results show that only about 5% of all sample/rsID combinations have high imputation error, suggesting that nearly half of the imputation errors in the ancient samples can likely be attributed to such reference error. On the other hand, our data also shows that, for the majority of imputed genotypes, the imputation error frequency caused by partial genotype information is consistently low, even when the genome coverage is as low as 0.5×.

The probabilities of each phenotype, including 3 eye colours, 7 hair and 11 skin tones, are based on 14 *p-values* calculated from the underlying 41 genetic markers of the HIrisPlex-S system. Since these probabilities are contingent upon the combination of multiple markers, it is expected that in most cases, the observed 1–2 imputation errors out of the 41 HIrisPlex-S markers do not significantly impact the phenotype probabilities and the derived phenotypic classifications. This is particularly true for intermediate phenotypes, where the p-values may shift slightly in either direction, but this typically does not result in substantially different phenotype probabilities and classifications. In principle, no or light pigment phenotypes such as blue eyes, blonde hair, and very pale skin are more susceptible to severe misclassification, as imputation error in any of the underlying homozygous “no pigment” state markers leads to a darker complexity phenotype being assigned. In the case of the 20 modern blue-eyed individuals, the prediction based on the imputed genotypes was concordant with the expectation in 95% of the cases at 0.5× genome coverage (Supplementary Table S1). Our results show that the majority of imputation replicates also properly classified the predicted ancient blue-eyed samples at 1× genome coverage or higher. However, at 0.5× genome coverage, three out of 5 ancient samples with blue eyes experienced higher misclassification rates compared to the expected classification (Supplementary Table S5) based on the high coverage pileup genotypes. Our findings revealed in these three ancient northern European samples that the primary cause of imputation errors and subsequent misclassification was the reference panel bias of the rs12913832 marker. Our results also revealed that the so-called opposite genotype (HOM REF instead of HOM ALT or vice versa) imputation errors are extremely rare both in the modern and ancient data. Consequently, the imputation errors on the predicted phenotypes are mostly causing a slight shift in the phenotype probabilities, often leading to similar predictions. Furthermore, while imputation errors equally impact the markers associated with darker shades, the heuristic applied in the phenotype classification scheme, which gives precedence to the darker tones, mitigates this issue; thus, the classification of individuals with very dark pigmentation is less severely affected. Ultimately, the 31 ancient samples were properly classified for the three phenotypic traits in the majority of parallel imputations, even at 0.1× genome coverages. The skin colour classification showed slightly larger phenotypic variability, but the predicted skin tones showed only minor differences compared to the truth (Supplementary Table S5). The higher incidence of observed minor differences in the indicated skin tone can likely be attributed to the more finely graded skin phenotype classification, which relies on a larger number of skin tone-associated genetic markers and involves more complex heuristic decision rules for deriving probabilities.

It is noteworthy that at 0.5× genome coverage, statistically, only half of the markers have pseudo-haploid genotype information available, and at 0.1× genome coverage, only 4–5 markers are covered by a single read. Despite significant missing information, the spatial genotype context flanking the 41 markers enabled proper imputation. The high concordance allowed the imputed genotypes from low-coverage aDNA sequences to be used with the validated HIrisPlex-S system, which requires the diploid genotypes for each of its 41 markers to predict phenotypes. The highly accurate phenotype predictions underscore the strength of imputation and demonstrate that even at these extremely low genome coverages, the applied method yields results comparable to phenotype predictions derived from the original high-coverage data.

## Conclusion

In summary, we have developed an easily deployable, user-friendly, imputation-based workflow that incorporates the validated HIrisPlex-S system for phenotyping ancient samples. The provided tools eliminate the tedious, error-prone manual translation between the reference-based NGS data and the probe-based HIrisPlex-S system allele count format, and it also allows the easy interpretation of the HIrisPlex-S p-values (based on the rules described in the HIrisPlex-S manual) to predict the most probable phenotypes. The proposed workflow is practical for analysing ancient whole-genome sequencing (WGS) data, even at low genome coverages of 0.1× to 0.5×, with the expectation of accurate classification in the majority of cases. Our results suggest that modern sequences with no genotyping errors and a closely matching reference lead to improved imputation accuracy, even when dealing with very low coverage. Currently, only a handful of ancient samples have been phenotyped due to the absence of suitable tools. Our workflow and the tools we’ve developed now enable the analysis of approximately 1500–2000 publicly accessible ancient WGS datasets that possess sufficient coverage to predict the visible phenotypic traits of past populations. We have to note that phenotyping ancient individuals, especially in the case of community-driven research where the aim is to uncover the recent history of specific populations, could have ethical concerns that have to be addressed. Since a significant portion of the challenges stems from reference panel bias, it is anticipated that as reference genomes become enriched with high-quality data (including ancient sequences), this workflow could achieve even higher concordance rates. Furthermore, as our understanding of genotype–phenotype associations continues to expand, the proposed workflow can be readily extended to accommodate new phenotypic markers. Additionally, aside from ancient samples, this framework can be employed for the analysis of degraded forensic samples, where the HIrisPlex-S multiple PCR-based method is hindered by short DNA fragments.

## Methods

### Selection of 1KG individuals for validation

To evaluate the accuracy of imputation of the 41 HIrisPlex-S markers on diverse individuals with different ancestry and phenotype traits based on validated genotype data, we used the genotypes of the 1000 Genome Project Phase 3 VCF data using the GRCh38 aligned data set. This data set contains 3202 individuals and more red hair individuals compared to the 2504 individuals of the original GRCh37 aligned phase III dataset. As imputation needs a reference data set first, we phenotyped all 2504 unrelated individuals with the HIrisPlex-S system to be able to select the most informative individuals. Next, we selected a subset of individuals for each 1 KG population that represented all of the phenotype traits present in the given population. Since red hair is caused by rare mutations in the MC1R gene, and imputation of low MAF variants are known to be less accurate, we filtered out all 1 KG individuals that had a mutation at any of the MC1R mutation loci included in the HIrisPlex-S kit. From these individuals, we included 8 additional individuals in our validation samples, each of them with a unique rare mutation (or different allele count) at different MC1R loci. Altogether, we selected 93 1 KG individuals and used their publicly available alignments to simulate low genome coverage. The ID, ancestry information, geno- and phenotype information of the selected individuals are shown in Supplementary Table S1.

### Selection of ancient individuals

We selected 31 publicly available, high-coverage ancient samples from the Allen Ancient Dataset (AADR, version V54.1)^17,28^. Specifically, we chose samples that underwent shotgun whole-genome sequencing (WGS) and had genome coverage exceeding 10×. The annotations of the 31 selected samples, including information about their origin and sample type, can be found in Supplementary Table S3. The selected samples include both UDG and non-UDG treated samples, originating from various geolocations (including samples with EUR and AFR ancestry), and span a wide range of dates from 41400BP to 1920CE. In all of our downstream analyses, we used the high coverage alignment files (BAM) available in public repositories referenced in the original manuscripts.

### Simulation of low coverage data

We computed the total average genome coverage for each high-coverage modern and ancient genome using mosdepth^39^. To simulate low genome coverage, we used ***samtools***^40^ with the appropriate “***view –subsample 0.FRAC –subsample-seed INT***” options to downsample the original high-coverage BAM files. From each high coverage BAM file, we generated ten parallel downsampled 2×, 1×, 0.5×, and 0.1× genome coverage data sets, each with different (seed 1–10) random seed.

### Preparation of reference marker sets

We prepared GRCh37, hg19, GRCh38, and hg38 reference data sets to analyse the 41 HIrisPlex-S markers. In the case of the GRCh37 reference set, the joint VCF of 2504 unrelated GRCh37 aligned Phase III dataset was used (https://ftp.1000genomes.ebi.ac.uk/vol1/ftp/release/20130502/). In the case of the GRCh38 and hg38 references, the joint VCF including the 3202 individuals were used (http://ftp.1000genomes.ebi.ac.uk/vol1/ftp/data_collections/1000G_2504_high_coverage/working/20201028_3202_phased/). Using the appropriate reference genome coordinates, we created a BED file that contains the marker positions for each reference data. Using “***bedtools slop***”^41^, we extended the genome windows around each marker by 2.5 M base pairs in both the 5’ and 3’ directions. Then, we merged the overlapping genome windows using “***bedtools merge***”. If the resulting genome window was smaller than 5 million base pairs in size (in the case of markers near telomeres or centromeres), we extended the genome window in the opposite direction. As a result, each of the resulting genome windows was at least 5 million base pairs, ensuring an ample number of flanking markers around the 41 HIrisPlex-S markers. We used the recommended options and workflow described in the GLIMPSE2 tutorial to prepare the binary reference data based on the appropriate 1 KG genotypes of the selected regions. We used GLIMPSE2_chunk with the “*–recursive*” option to create a list of genome regions suitable for imputation. We used the biallelic SNPs of the appropriate 1 KG Phase 3 reference data. As the GLIMPSE2 framework only imputes genotypes at positions that exist in the reference data, we manually included the genotypes of the single biallelic dupA variant for red hair (rs312262906) to the reference. GLIMPSE2 can work with any biallelic variants in the reference data and is not restricted that the reference haplotype catalogues have to contain only SNPs. The GLIMPSE2_split_reference tool was used to generate the binary reference files required to impute the variants in the 11 genome regions containing the 41 HIrisPlex-S markers.

### Detailed description of the aHISplex tool and workflow

We created an easily deployable package (https://github.com/zmaroti/aHISplex) that contains all the required tools, customised reference data and translation tables for the different reference genomes to run the whole analysis workflow from aligned BAM file(s) to phenotype classification. The workflow consists of three steps.

- The first step is to run the GLIMPSE2-based imputation on a single BAM file or list of BAM files and filter/translate the imputed genotypes of the HIrisPlex-S markers, resulting in a HIrisPlex-S web tool compatible data file. We used the default parameters recommended in the GLIMPSE2 tutorial. The included tool saves all the underlying raw analysis files including the unmerged imputed/phased bcf output and the log files of GLIMPSE2 phasing and ligation.
- In the second step, the ‘*HISplex41_upload.csv*’ output file is generated using the ‘bcftools query’ command with the appropriate position files to filter the 41 HIrsisPlex-S marker genotypes. The genotypes are translated from the reference-based genotypes to the probe-based HIrisPlex-S upload data file format using the included ***transToHISplex*** with the appropriate translation tables. The resulting file has to be uploaded and analysed at the official https://hirisplex.erasmusmc.nl/ website using the HIrisPlex-S batch upload phenotyping web service.
- In the third step, the downloaded results file can be processed by the ***classifHISplex*** tool (included in our software package) to evaluate the resulting phenotype probabilities and classify each analysed sample for the three phenotypic traits based on the rules described in the HIrisPlex-S manual^11^.

The tools of the aHISplex software package are written in golang, and a shell script is included as a glue to call and run the public (GLIMPSE2_phase, GLIMPSE2_ligate, bcftools and optionally GNU parallel tool) and the included **transToHISplex** tool with the appropriate parameters. The whole analysis workflow (except the web tool part) can be performed with two commands, providing the BAM file(s) and applying the included ***classifHISplextool*** to interpret the output of the web tool. The github page contains a detailed README for the dependencies, installation, and usage of the package.

### Assessment of aHISplex performance on the HIrisPlex-S system

We downsampled each sample for each genome coverage in ten parallel runs with different (1–10) random seeds. Thus, the available genotype information (how many reads and at which ratio the alleles are represented) for the imputed markers was different in each downsampled data set. In the case of ancient data, the postmortem damage leads to a variable rate of genotyping errors. Since genotyping errors could influence the posterior haplotype probabilities, multiple different solutions may exist (with different probabilities), leading to different imputed genotypes even from the same sequence information. To ensure that due to post mortem damage of ancient samples the imputation still results in very similar haplotype probabilities, we performed ten different imputations using each ten parallel downsampled data for each genome coverage and ancient sample, resulting in 10 (imputation parallels) * 10 (downsampled parallels) * 5 (different genome coverages) analysis for each of the 31 analysed sample.

For modern samples, we used the 1 KG Phase 3 genotype data (from the VCF files) as truth (Supplementary Table S1). In the case of ancient samples, we genotyped the high-coverage ancient BAM files based on the allele pileup (***samtools mpileup***) at genome positions of the 41 HIrisPlex-S markers (Supplementary Table S4). Using the genotypes, the strand and test allele information of the HIrisPlex-S markers, we translated the reference-based genotype information to the allele counts for each marker and sample and used this information as ground truth for the assessment of the imputation genotype concordance.

For the imputation-based genotype predictions, we calculated the genotype concordance against the ground truth. We counted total differences, including all genotypes that were imputed differently, and we also counted the gross error situation, where an HOM ALT truth was imputed as HOM REF (or the opposite). Furthermore, since imputation can result in different genotypes from the same input data, we also calculated the count/frequency of minor alleles (imputation variability).

Using the imputation-based allele counts of the 41 HIrisPlex-S markers for each sample at each coverage and parallels, we calculated the phenotype probability calculation using the HIrisPlex web server to obtain the probability scores of the predicted hair, eye, and skin colours (Supplementary Table S5). We used the included ***classifHISplex*** tool to translate the phenotype probabilities to eye, hair and skin colour shades based on the rules defined in the “HIRISPLEX-S, HIRISPLEX & IRISPLEX Eye, Hair and Skin colour DNA Phenotyping web tool USER MANUAL”^11^.

## Supplementary Information

Below is the link to the electronic supplementary material.

Supplementary Material 1

Supplementary Material 2

Supplementary Material 3

Supplementary Material 4

Supplementary Material 5

Supplementary Material 6

Supplementary Material 7

Supplementary Material 8

## Data Availability

We used publicly available data for the validation of the method. Our software, with potential future updates, is available at the GitHub repository (https://github.com/zmaroti/aHISplex).
